# Soluble Whey Protein Hydrolysate Ameliorates Muscle Atrophy Induced by Immobilization via Regulating the PI3K/Akt Pathway in C57BL/6 Mice

**DOI:** 10.3390/nu12113362

**Published:** 2020-11-01

**Authors:** Ji Eun Shin, Seok Jun Park, Seung Il Ahn, Se-Young Choung

**Affiliations:** 1 Department of Life and Nanopharmaceutical Sciences, Graduate School, Kyung Hee University, Seoul 02447, Korea; cindy@khu.ac.kr; 2 Health & Nutrition R&D Group, Maeil Dairies Co., Ltd., Gyeonggi 17714, Korea; sj.park@maeil.com; 3 Neocremar R&D Center, 211, Jungdae-ro, Songpa-gu, Seoul 05702, Korea; seungil@cremar.co.kr; 4 Department of Preventive Pharmacy and Toxicology, College of Pharmacy, Kyung Hee University, Seoul 02447, Korea

**Keywords:** whey protein hydrolysate, muscle atrophy, sarcopenia, immobilization, muscle synthesis, muscle degradation, PI3K/Akt pathway

## Abstract

Sarcopenia, a loss of skeletal muscle mass and function, is prevalent in older people and associated with functional decline and mortality. Protein supplementation is necessary to maintain skeletal muscle mass and whey protein hydrolysates have the best nutrient quality among food proteins. In the first study, C57BL/6 mice were subjected to immobilization for 1 week to induce muscle atrophy. Then, mice were administered with four different whey protein hydrolysates for 2 weeks with continuous immobilization. Among them, soluble whey protein hydrolysate (WP-S) had the greatest increase in grip strength, muscle weight, and cross-sectional area of muscle fiber than other whey protein hydrolysates. To investigate the molecular mechanism, we conducted another experiment with the same experimental design. WP-S significantly promoted the phosphoinositide 3-kinase (PI3K)/protein kinase B (Akt)/mammalian target of rapamycin (mTOR) pathway and inhibited the PI3K/Akt/forkhead box O (FoxO) pathway. In addition, it increased myosin heavy chain (MyHC) expression in both the soleus and quadriceps and changed MyHC isoform expressions. In conclusion, WP-S attenuated muscle atrophy induced by immobilization by enhancing the net protein content regulating muscle protein synthesis and degradation. Thus, it is a necessary and probable candidate for developing functional food to prevent sarcopenia.

## 1. Introduction

Skeletal muscle is the largest organ that constitutes approximately 40% of total body weight and conducts many important functions in the body. It enables individuals to move, supports the skeleton and organs, and regulates glucose homeostasis [1]. Thus, the loss of muscle mass causes discomfort in daily life and, even worse, exacerbates other metabolic diseases [2]. Skeletal muscle mass is regulated by the balance between muscle protein synthesis (MPS) and muscle protein degradation (MPD) [3]. A loss of muscle mass occurs when this balance is broken (MPS < MPD) and it can lead to muscle atrophy [4]. A continuous state of muscle atrophy and a decrease in muscle strength are now defined as sarcopenia. Sarcopenia is prevalent in older people and is associated with health and functional decline, disease and injury, quality of life, and even mortality.

Older people tend to eat less protein than younger people. According to a recent study, about 10 to 30% of community-dwelling old people do not take the estimated average requirement for daily protein intake (0.7 g/kg/day), which is necessary to maintain skeletal muscle mass [5]. Among food proteins, whey protein has good nutrition quality. It has the highest amino acid content (essential amino acids and branched amino acids (BCAA)) compared with plant- and animal-based protein sources [6,7].

Whey protein is a mixture of proteins isolated from byproducts of cheese production. There are two types of whey: sweet whey and acid whey. Sweet whey is a byproduct resulting from the manufacture of cheese coagulated with rennet. Since most cheeses are made using rennet, sweet whey is generally expressed as whey. Acid whey is a byproduct resulting from the production of cheese coagulated by acids. Whey protein contains lactoferrin, β-lactoglobulin, α-lactalbumin, glycomacropeptide, and immunoglobulins [8]. Whey protein is an important nutrient for muscle metabolism because it increases the rate of muscle protein synthesis and decreases that of muscle protein degradation [7,9,10]. Whey protein hydrolysate, a hydrolyzed form of whey protein, is rapidly absorbed and digested into free amino acids into plasma and induces skeletal muscle protein anabolism. Morifuji et al. demonstrated that the intake of whey protein hydrolysates increases plasma amino acid concentration more than intact whey protein intake dose [11]. In addition, whey protein and whey protein hydrolysate have a high amount of BCAA. Many studies suggested that BCAA, especially leucine, stimulate MPS through activating the mTOR [12,13,14].

Muscle protein synthesis is mainly regulated by the PI3K/Akt signaling pathway [15,16,17]. When insulin or insulin-like growth factor 1 (IGF-1) is secreted and attached to its receptor in muscle, it phosphorylates the insulin receptor substrate 1, subsequently activating PI3K. PI3K turns phosphatidylinositol 4,5-bisphosphate into phosphatidylinositol 3,4,5-triphosphate in the membrane, accelerates the adhesion of Akt, and phosphorylates it. Akt activates the mTOR which exists as mTOR complex 1 (mTORC1). mTORC1 regulates translation through the phosphorylation of substrates such as eukaryotic translation initiation factor 4E-binding protein 1 (4E-BP1) and ribosomal protein S6 kinase beta-1 (S6K1). S6K1 and 4E-BP1 promote muscle protein synthesis by activating ribosomal protein S6 and releasing the translation initiation factor eIF4E, respectively. Akt also inhibits FoxO, a transcription factor of atrogenes, through the phosphorylation, resulting in the translocation of FoxO from the nucleus to the cytoplasm [18].

Muscle protein degradation is mostly affected by the ubiquitin–proteasome system and the autophagosome–lysosome system. FoxO controls these two muscle protein degradation systems by regulating the expressions of many atrogenes [19]. Two muscle-specific E3 ligases, muscle atrophy F-box protein (Atrogin-1) and muscle ring finger-1 (MuRF1), have been widely used as markers for ubiquitin–proteasome activity in many muscle atrophy models [20,21]. Mechanisms of autophagy are still unknown but a study found that overexpression of BCL2/adenovirus E1B 19 kDa protein-interacting protein 3 (Bnip3) is able to induce autophagy, whereas Bnip3 knockdown reduces FoxO3-induced autophagy in vivo [22].

Both muscle protein synthesis and degradation are sensitive to stimuli such as nutrients, exercise, hormones, and disease state [23]. Different strategies have been indicated to reduce muscle atrophy such as protein supplementation, resistance or endurance exercise, and antioxidant/anti-inflammatory compounds [24]. Among them, we focused on the effect of protein supplementation, especially whey protein hydrolysate. Although whey protein hydrolysate has been widely studied concerning its anabolic effects on muscle [25,26,27], there were no studies comparing hydrolysis conditions to find the best hydrolysate or investigating its efficacy in muscle atrophic states. We hydrolyzed whey protein concentrate or acid whey into four different whey protein hydrolysates and compared their effects on muscle atrophy. Based on the results, we selected the most effective whey protein hydrolysate against muscle atrophy. Then, we conducted another experiment using the selected sample with three dosages to study the optimal dose to use in clinical trials and investigated its molecular mechanism.

## 2. Materials and Methods

### 2.1. Preparation of Whey Protein Hydrolysates

Acid whey or whey protein concentrate (WPC) was dissolved in distilled water (50~55 °C) to become 20% (*w/w*). The solution was adjusted to become pH 7 to 7.5 using sodium bicarbonate. After, 0.2% Alcalase 2.4 L FG (Novozyme, Denmark) and 0.2% Protamex (Novozyme, Denmark) were added into the solution and incubated for 4 h at 50~55 °C. Then, 0.2% Flavourzyme 1000 L (Novozyme, Denmark) was added and incubated for 15 h at 50~55 °C. After the enzyme reactions, the solution was inactivated by boiling at 90 °C for 10 min. The inactivated solution was filtered using a 1μm filter paper to remove unhydrolyzed WPC or acid whey. Then, they were heat sterilized and dried using a spray dryer.

Acid whey that had undergone the above method, except for the filtration, was named whey acidic protein hydrolysate (AW-H). Acid whey that had undergone the above method was named soluble whey acidic protein hydrolysate (AW-S). WPC that had undergone the above method, except for the filtration, was named whey protein hydrolysate (WP-H). WPC that had undergone the above method was named soluble whey protein hydrolysate (WP-S). All the samples were provided by Neocremar Co.,Ltd. (Seoul, Korea). These preparation methods are summarized in Supplementary Figure S1.

### 2.2. Amino Acid Composition Analysis

In a cap tube, 25 mg AW-H, AW-S, WP-H, and WP-S were weighed and 2.5 mL 6N HCl were added. Then, they were hydrolyzed for 24 h at 110 °C. The unhydrolyzed materials were removed by the 3G-4 glass filter and the filtrates were completely volatilized using a rotary evaporator (N-1110, EYELA, Tokyo, Japan) at 50 °C. After volatilization, they were used as a sample for analysis, adding 0.01N HCl, and were analyzed using an automatic analyzer (Biochrom 30, Cambridge, UK). The results are shown in Table 1.

### 2.3. Animals and Experimental Design

Male C57BL/6 mice (5-weeks-old) were purchased from Raon Bio (Yongin, Korea) and housed in a standard animal facility maintained on a 12 h:12 h light–dark cycle at 25 ± 1 °C with free to access food (Teklad Global 18% Protein Rodent Diet, ENVIGO, Indianapolis, IN, USA) and water. The protocol of the animal study was approved by the Institutional Animal Care and Use Committee guidelines of Kyung Hee University and the approval number was KHUASP(SE)-19-251.

In the first study, mice were randomly divided into a normal group (n = 7) and an immobilization (IM) group (n = 35). The IM group was subjected to hindlimb immobilization using a 1.5 mL microfuge tube, metal paper clip, and velcro loop [28]. After 1 week of IM to induce muscle atrophy, the IM group was randomly divided into 5 groups (an immobilization group (IM) and sample treatment groups (AW-H, AW-S, WP-H, and WP-S). Treatment groups were orally administered with four different whey protein hydrolysates (AW-H, AW-S, WP-H, and WP-S) for 2 weeks with continuous IM.

In the second study, mice were randomly divided into a normal group (n = 8) and an IM group (n = 32). The IM group was subjected to hindlimb immobilization with the above method. After 1 week of IM, the IM group was randomly divided into 4 groups (an immobilization group (IM) or WP-S treated groups (WP-S 400, WP-S 800, and WP-S 1200)). WP-S treated groups were orally administered with WP-S for 2 weeks with continuous IM. The dosages for WP-S-treated groups were 400 mg/kg (WP-S 400 group), 800 mg/kg (WP-S 800 group), and 1200 mg/kg (WP-S 1200 group).

Whey protein hydrolysate has been widely studied concerning its anabolic effects on muscle [25,26,27]. Most of the studies compared the differences between a normal group and whey protein hydrolysate administration groups. In addition, as a developing process for functional food about sarcopenia, we wanted to focus on the effects of whey protein hydrolysate in the muscle atrophy model. Thus, we did not consider adding a control group (non-immobilized + whey protein hydrolysate administration) because it has been widely studied [27,29,30,31] and we did not target a healthy person.

The experimental design is expressed in Supplementary Figure S2; the nutrient contents of diet are shown in Supplementary Table S1. WP-S nutrient contents are also shown in Table 2.

### 2.4. Dosage Information

Mice were administered daily with whey protein hydrolysates for 2 weeks by gavage feeding. In the first study, the dosages were all 800 mg/kg, which is equivalent to about 4 g/day in a 60 kg human (conversion factor is 12.3) [32]. In the second study, the doses were 400, 800, and 1200 mg/kg (equivalent in a 60 kg human: 2, 4, and 6 g/day). The total protein intake was calculated adding protein intake amounts from an 18% protein-containing diet and whey protein hydrolysates administration ((0.18 × food intake (g) × 1000) + (whey protein hydrolysates intake dose (mg/kg) × body weight (g)/1000)). This is shown in Supplementary Figure S3.

### 2.5. Measurement of Grip Strength

Grip strength was measured every 3 days during the animal experiment using a grip strength test (Bioseb, Chaville, France). Mice were lifted by the tail and placed on the grid connected with the grip strength test [33]. After the mice held the grid, we pulled them horizontally until the grip was broken. The maximum force of grip was measured and we used the average of five measurements for analysis. The grip strength was normalized to body weight [34].

### 2.6. The Toxicity Test of WP-S

To confirm the safety of WP-S administration, we investigated Alanine aminotransferase (ALT) and blood urea nitrogen (BUN) levels in serum. At the end of the experiment, mice were anesthetized with isoflurane gas, and blood samples were collected from the inferior vena cava. The blood samples were incubated at room temperature for 30 min, and the serum was obtained after centrifuging the blood at 3000× *g* rpm for 15 min at 4 °C. ALT and BUN levels were measured using commercial kits purchased from Asan Diagnostics (Seoul, Korea) following the manufacturer’s instructions. Results were shown in the Supplementary Figure S6.

### 2.7. Measurement of Muscle Weight and Protein Content

After sacrifice, the quadriceps, gastrocnemius, and soleus were collected from the immobilized hindlimb and weighed. The gastrocnemius was homogenized with liquid nitrogen and lysed using a lysis buffer containing cOmplete™ Protease Inhibitor Cocktail and PhosSTOP™ (Roche Diagnostics, Indianapolis, IN, USA). Then, the lysates were centrifuged (13,000× *g* rpm, 15 min, 4 °C) and the supernatants were collected. The protein content was measured using a Pierce™ BCA Protein Assay Kit (Thermo Fisher Scientific, Rockford, IL, USA) following the manufacturer’s instructions.

### 2.8. Histological Analysis of Muscle Cross-Sectional Area (CSA)

After sacrifice, the gastrocnemius was obtained to conduct histological analysis. Gastrocnemius was fixed with 4% paraformaldehyde and sliced into 4 μm-thick paraffin-embedded sections. Then, the sections were stained with hematoxylin and eosin (H&E) for 13 h. Stained sections were visualized using an optical microscope (Olympus, Tokyo, Japan). The representative images (100×) of stained sections per group (n = 6) were used. Then, we quantified the CSA of about 30–40 myofibers in each image using Image J software (National Institute of Health, Bethesda, MD, USA). Measurement was done from the largest myofibers to smaller ones.

### 2.9. Quantitative Real Time-PCR (qRT-PCR) Assay

Twenty milligrams of gastrocnemius or quadriceps and total soleus were homogenized with a liquid nitrogen and total RNA was extracted using easy-RED™ (iNtRON, Seongnam, Korea) according to the manufacturer’s protocol. The quantity and purity of extracted RNA were measured using a NanoDrop ND-1000 spectrophotometer (Thermo Scientific, Gaithersburg, MD, USA) [35]. Then, cDNA was synthesized from the extracted RNA using a PrimeScript™ 1st strand cDNA Synthesis Kit (TaKaRa, Tokyo, Japan). qRT-PCR was performed using a Step One Plus™ Real-Time PCR System (Applied Biosystems, Foster City, CA, USA) with TB Green™ Premix Ex Taq™ (TaKaRa, Tokyo, Japan). The primer sequence is listed in Table 3. The mRNA levels were normalized to the *Gapdh* and calculated using the comparative method (2^−ΔΔCt^).

### 2.10. Western Blot Assay

The muscle tissue (gastrocnemius or quadriceps) was homogenized with liquid nitrogen and lysed using a lysis buffer. Then, the lysates were centrifuged and the supernatants were collected. The concentrations of protein were measured using a commercial kit. These processes are the same as 2.6. The same amount of protein was loaded on a 12% or 15% polyacrylamide gel and was transferred to a polyvinylidene fluoride membrane. Then, the membrane was blocked by 5% skim milk and incubated overnight with a primary antibody (diluted as 1:1000 with BSA). The primary antibodies were purchased from Cell Signaling (MA, USA) (p-PI3K (#4228), Akt (#9272), p-Akt (#9271), mTOR (#2972), p-mTOR (#2971), S6K1 (#9202), p-S6K1 (#9205), 4E-BP1 (#9452), p-4E-BP1 (#2855), FoxO3a (#12829), p-FoxO3a (#9465), and BNIP3 (#3769)), Abcam (Cambridge, UK) (PI3K (ab191606)), Sigma-Aldrich (MO, USA) (p62 (P0067)), GeneTex (CA, USA) (β-actin (GT5512) and GAPDH (GT239)), and Santa Cruz Biotechnology (CA, USA) (Atrogin-1 (sc-166806) and MurF1 (sc-398608)). The next day, it was incubated with a horseradish peroxidase-conjugated secondary antibody (GeneTex, CA, USA) for 100 m (diluted from 1:600 to 1: 2000 with 5% skim milk). Then, it was visualized using a LAS3000 luminescent image analyzer (Fuji Film, Tokyo, Japan). The protein expression level was analyzed using the Image J software (National Institute of Health, MD, USA) and normalized to the β-actin or GAPDH.

### 2.11. Statistical Analysis

The results were expressed as the mean ± SD. The Shapiro–Wilk normality test was conducted to verify the normality of the data. The normally distributed data were analyzed using one-way ANOVA followed by Tukey’s post hoc test. Statistical significance was determined by SPSS version 25 statistical software (Chicago, IL, USA) and it was expressed as follows: ^#^ *p* < 0.05, ^##^ *p* < 0.01, and ^###^ *p* < 0.001 compared to the normal. * *p* < 0.05, ** *p* < 0.01, and *** *p* < 0.001 compared to the IM.

## 3. Results

### 3.1. Comparison of Four Different Whey Protein Hydrolysates (AW-H, AW-S, WP-H, and WP-S) in Immobilization-Induced Muscle Atrophy Model

**Figure 1:**
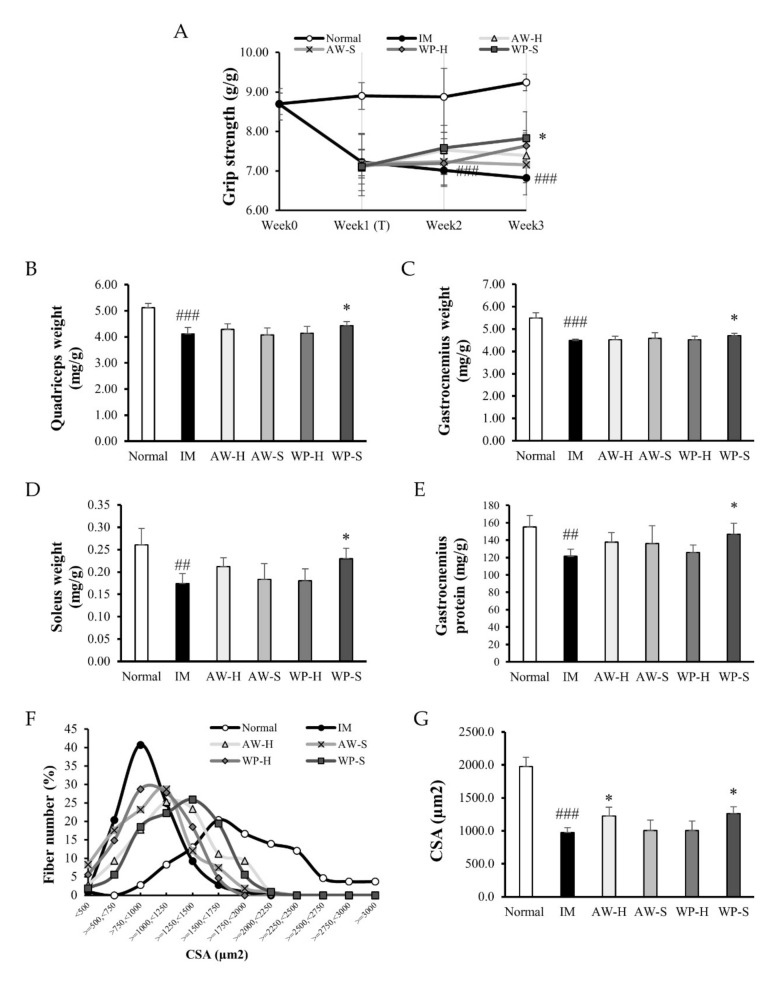
Comparison of four different whey protein hydrolysates (AW-H, AW-S, WP-H, and WP-S) in an immobilization-induced muscle atrophy model. C57BL/6 mice were subjected to hindlimb immobilization for 1 week (except for normal group) and then, were administered with AW-H, AW-S, WP-H, or WP-S for an additional 2 weeks with continuous immobilization. (**A**) The grip strength during an animal experiment. It was expressed as a ratio to body weight (g/g). (**B**–**D**) The skeletal muscle weight in three different muscle tissues (quadriceps, gastrocnemius, and soleus). It was shown as a ratio to the body weight (mg/g). (**E**) The total gastrocnemius protein content. It was shown as a ratio to the gastrocnemius weight (mg/g). (**F**) The distribution graph of muscle fiber cross-sectional area (CSA) and (**G**) The average CSA in each muscle fiber. They were measured using H&E-stained slides of gastrocnemius. Data are expressed as mean ± SD. ^##^ *p* < 0.01, ^###^ *p* < 0.001 versus normal. * *p* < 0.05 versus IM.

The total protein intake did not show a significant difference compared with the IM group (except for the AW-S group), so we could interpret that the results of further experiments are caused by the administration of four different whey protein hydrolysates itself (Supplementary Figure S3). Body weight after the experiment did not show a significant difference (Supplementary Figure S4). One week of IM significantly decreased grip strength and showed the onset of muscle atrophy by IM. At the end of the experiment, grip strength was decreased by 26.2% in the IM group compared to the normal group and it was significantly increased by 14.7% in the WP-S group (Figure 1A).

After sacrifice, we dissected three different skeletal muscle tissue (fast-twitch muscle: quadriceps and gastrocnemius; slow-twitch muscle: soleus). The muscle masses of skeletal muscle (quadriceps, gastrocnemius, and soleus) were significantly decreased by 19.7%, 18.2%, and 33.2% in the IM group compared to the normal group. They were significantly increased by 7.8%, 4.7%, and 32.0% in the WP-S group compared to the IM group (Figure 1B–1D). The total gastrocnemius protein content was significantly decreased by 22% in the IM group versus the normal group. It was significantly increased by 21% in the WP-S group compared to the IM group (Figure 1E). The histological analysis was conducted by measuring the cross-sectional area (CSA) of each muscle fiber (the representative images were shown in Supplementary Figure S5). In Figure 1F, the CSA distribution of each fiber in the IM group was decreased. Additionally, the CSA distribution of each fiber was enlarged by the whey protein hydrolysate treatment (WP-H = AW-S < AW-H < WP-S). In addition, the mean CSA was 50.6% lower in the IM group than the normal group. It was significantly increased by 25.6% in the AW-H group and by 29.5% in the WP-S group than the IM group (Figure 1G).

### 3.2. Comparison of the Efficacy of Four Different Whey Protein Hydrolysates on Muscle Protein Synthesis and Degradation

**Figure 2:**
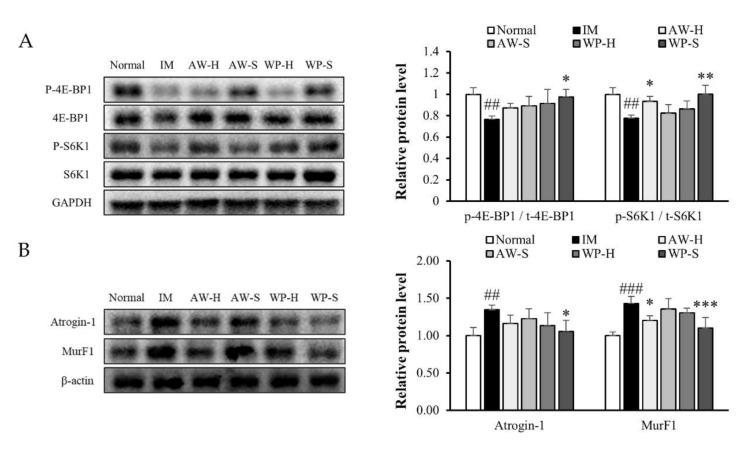
Comparison of the efficacy of four different whey protein hydrolysates on muscle protein synthesis and degradation. C57BL/6 mice were subjected to hindlimb immobilization for 1 week (except for normal group) and then, were administered with AW-H, AW-S, WP-H, or WP-S for an additional 2 weeks with continuous immobilization. (**A**) The phosphorylation of 4E-BP1 and S6K1 in gastrocnemius. The protein levels were normalized to the GAPDH. (**B**) The protein expression of Atrogin-1 and MurF1 in gastrocnemius. They were normalized to the β-actin. Data are expressed as mean ± SD (n = 4 or 5 per group). ^##^ *p* < 0.01, ^###^ *p* < 0.001 versus normal. * *p* < 0.05, ** *p* < 0.01, *** *p* < 0.001 versus IM.

We investigated some proteins involved in muscle protein synthesis and degradation. The phosphorylation ratios of 4E-BP1 and S6K1, which are known as markers of muscle protein synthesis, were measured. The phosphorylation ratio of 4E-BP1 was significantly declined in the IM group compared to the normal group and significantly raised in the WP-S group. The phosphorylation ratio of S6K1 was significantly decreased in the IM group with respect to the normal group and significantly increased in both the AW-H and WP-S group with respect to the IM group (Figure 2A). Protein expression levels of Atrogin-1 and MurF1, markers for muscle protein degradation, were significantly increased in the IM group compared to the normal group and these increases significantly decreased when treated with WP-S (Figure 2B).

### 3.3. WP-S Increased Grip Strength, Muscle Mass, and Cross-Sectional Area of Muscle Fiber

We conducted another study to investigate the molecular mechanism of WP-S using the same experimental design in Section 3.1 and administered three dosages of WP-S (400, 800, and 1200 mg/kg) to mice.

**Figure 3:**
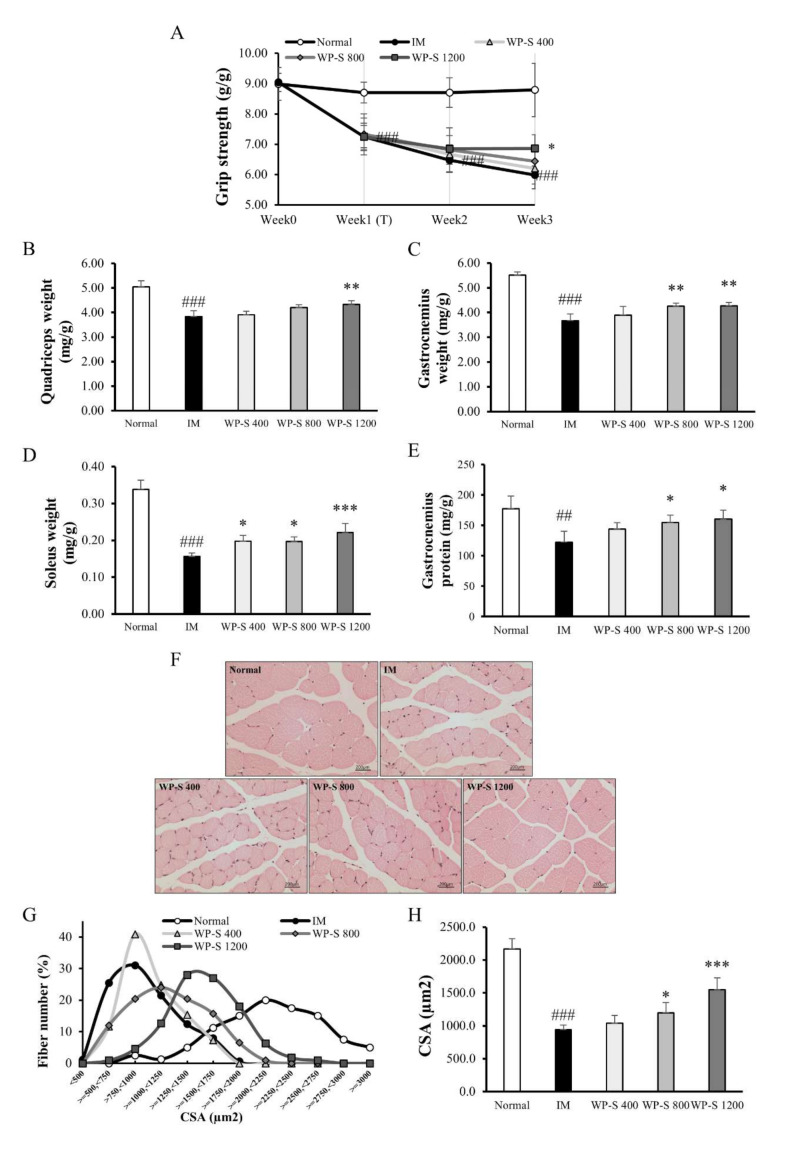
Effect of soluble whey protein hydrolysate (WP-S) in immobilization-induced muscle atrophy model. C57BL/6 mice were subjected to hindlimb immobilization for 1 week (except for normal group) and then, were administered with WP-S at 400, 800, or 1200 mg/kg for an additional 2 weeks with continuous immobilization. (**A**) The grip strength during the animal experiment. It was expressed as a ratio to the body weight (g/g). (**B**–**D**) The muscle mass of three muscle tissues (quadriceps, gastrocnemius, and soleus). It was expressed as a ratio to the body weight (mg/g). (**E**) The total gastrocnemius protein content. It was shown as a ratio to the gastrocnemius weight (mg/g). (**F**) The representative images of H&E stained sections of gastrocnemius (400x, scale bar = 200 μm). (**G**) The distribution graph of the muscle fiber cross-sectional area (CSA) and (**H**) The mean CSA of each muscle fiber. The results are expressed as mean ± SD. ^##^ *p* <0.01, ^###^ *p* < 0.001 versus normal. * *p* < 0.05, ** *p* < 0.01, *** *p* < 0.001 versus IM.

The total protein intake in the WP-S treatment groups did not show a significant difference compared to the IM group (Supplementary Figure S3). The body weights in the IM group and WP-S treatment groups were significantly decreased by about 7% compared to the normal group (Supplementary Figure S4). At the end of the experiment, the grip strength was significantly decreased 31.9% in the IM group than in the normal group and significantly increased by 14.6% in the WP-S 1200 group than in the IM group (Figure 3A).

The muscle weights (quadriceps, gastrocnemius, and soleus) were significantly decreased by 24.1%, 33.3%, and 53.9% in the IM group compared with the normal group. The quadriceps weight in the WP-S 1200 group was significantly increased by 12.8% versus the IM group. The gastrocnemius weights in the WP-S 800 and 1200 groups were significantly raised by 15.8% and 16.3% compared with the IM group. The soleus weights in the WP-S 400, 800, and 1200 groups were significantly enlarged by 26.6%, 26.1%, and 41.6% compared to the IM group (Figure 3B–D). The total gastrocnemius protein content was significantly decreased by 29% in the IM group versus the normal group. It was significantly increased by 23% and 27% in the WP-S 800 and 1200 groups compared to the IM group (Figure 3E).

The histological analysis showed that IM decreased the CSA of each fiber and WP-S treatment increased that in a dose-dependent manner (Figure 3F). The distribution graph showed that IM decreased the overall CSA of each fiber and WP-S treatment enlarged it dose-dependently (Figure 3G). The average CSA was significantly decreased in the IM group by 56.6% than in the normal group and was significantly increased by 27.6% and 64.8% in the WP-S 800 and 1200 groups than the IM group (Figure 3H).

### 3.4. WP-S Recovered the Imbalance between Muscle Protein Synthesis and Degradation

**Figure 4:**
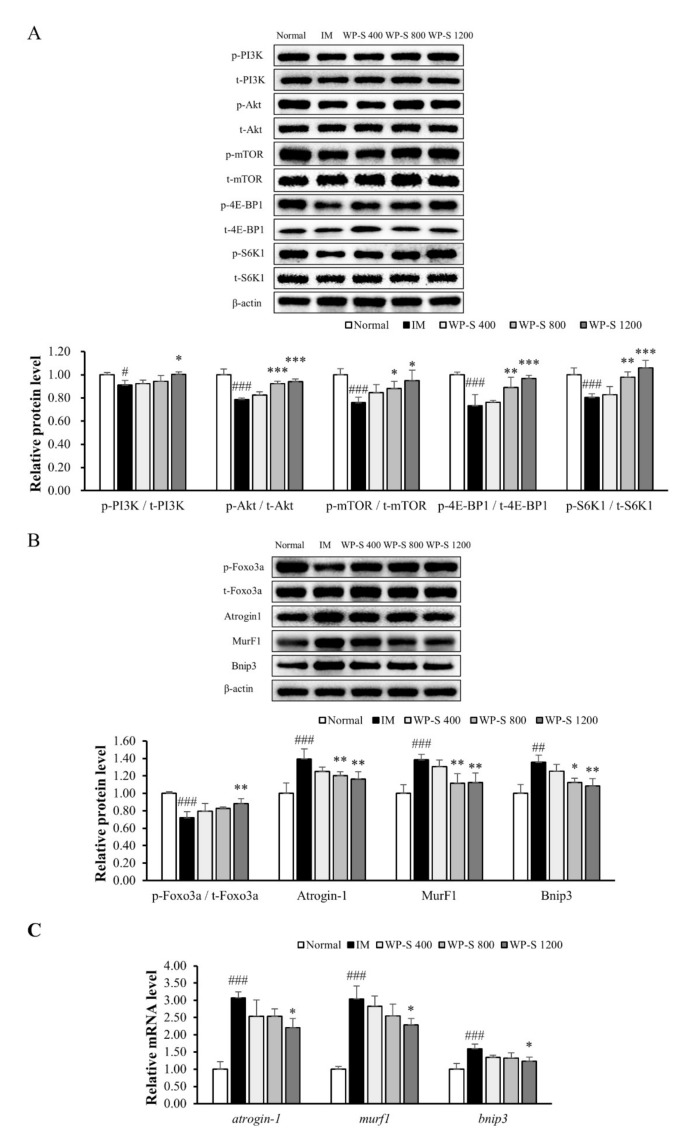
Effect of soluble whey protein hydrolysate (WP-S) on muscle protein synthesis and degradation. C57BL/6 mice were subjected to hindlimb immobilization for 1 week (except for normal group) and then, were administered with WP-S at 400, 800, or 1200 mg/kg for an additional 2 weeks with continuous immobilization. (**A**) The phosphorylation of factors associated with muscle protein synthesis in gastrocnemius and quadriceps. The protein levels were normalized to the β-actin. (**B**) The protein expressions related to muscle protein degradation in gastrocnemius. They were normalized to the β-actin. (**C**) The gene expressions related to muscle protein degradation. They were normalized to the *Gapdh*. The results are expressed as mean ± SD (n = 5 or 6 per group). ^#^ *p* < 0.05, ^##^ *p* < 0.01, ^###^ *p* < 0.001 versus normal. * *p* < 0.05, ** *p* < 0.01, *** *p* < 0.001 versus IM.

We investigated the effect of WP-S on the PI3K/Akt pathway, which is known to regulate muscle protein synthesis. The phosphorylation ratio of PI3K was significantly decreased in the IM group, followed by significant decreases in the phosphorylation ratio of Akt, mTOR, 4E-BP1, and S6K1. The WP-S administration attenuated these decreases induced by IM in a dose-dependent manner and, in the WP-S 1200 group, they were recovered to the normal level (Figure 4A).

We measured the expression of FoxO3a, which is a transcription factor that regulates the expression of *atrogin-1*, *murF1*, and *bnip3* and examined the protein and gene expression of Atrogin-1, MurF1, and Bnip3. The phosphorylation ratio of FoxO3a was significantly decreased in the IM group. WPS administration significantly increased it in a dose-dependent manner. When FoxO3a phosphorylates, it is translocated to cytoplasm and degraded; thus, the expression of Atrogin-1, MurF1, and Bnip3 declines. This assumption was observed in both protein and gene expression. The protein expression levels of Atrogin-1, MurF1, and Bnip3 were significantly decreased in the IM group and were increased by WP-S treatment in a dose-dependent manner (Figure 4B). In addition, the gene expression levels of *atrogin-1*, *murf1*, and *bnip3* were significantly decreased in the IM group and were increased in the WP-S-treated groups dose-dependently (Figure 4C).

### 3.5. WP-S Changed Total Myosin Heavy Chain and Its Isoform Expressions

**Figure 5:**
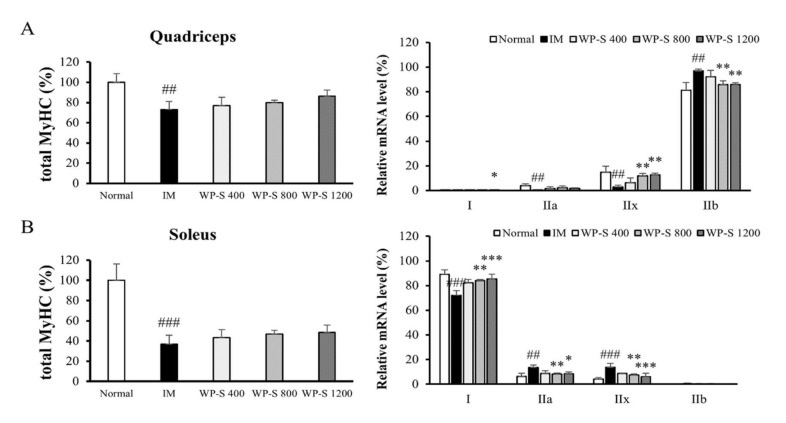
Effect of soluble whey protein hydrolysate on the myosin heavy chain (MyHC) expression. C57BL/6 mice were subjected to hindlimb immobilization for 1 week (except for normal group) and then were administered with WP-S at 400, 800, or 1200 mg/kg for an additional 2 weeks with continuous immobilization. The total MyHC was calculated, adding all MyHC isoform expression levels (MyHC I + MyHC IIa + MyHC IIx + MyHC IIb). (**A**) The changes in the expression of total MyHC and MyHC isoforms in quadriceps (fast-twitch muscle). (**B**) The alterations in the expression of total MyHC and MyHC isoforms in soleus (slow-twitch muscle). Data are expressed as mean ± SD (n = 5 per group). ^##^ *p* < 0.01, ^###^ *p* < 0.001 versus normal. * *p* < 0.05, ** *p* < 0.01, *** *p* < 0.001 versus IM.

To study the change of myosin heavy chain (MyHC) in two different muscle types, mRNA levels in the quadriceps and soleus were analyzed by qRT-PCR. The total MyHC expressions of both muscle tissues were significantly decreased in the IM group and were dose-dependently increased by WP-S administration (Figure 5A,B). In quadriceps, fast-twitch muscle, MyHC IIx, and IIb accounted for the majority of the MyHC isoform expressions [36]. The expression levels of MyHC IIa and IIx were significantly decreased and the expression level of MyHC IIb was significantly increased in the IM group. Considering that total MyHC significantly decreased in the IM group, it shows IM-induced decrease in MyHC expression level is more likely to hit slow MyHC isoforms than IIb. These changes were recovered by WP-S administration in a dose-dependent manner (Figure 5A).

The other way, in soleus (slow-twitch muscle), MyHC I occupied the majority of the MyHC isoform expressions [36]. In the IM group, the expression level of MyHC I was significantly down-regulated and the expression levels of MyHC IIa and IIx were significantly up-regulated. These tendencies were reversed by the WP-S treatment dose-dependently (Figure 5B).

## 4. Discussion

Sarcopenia occurs due to muscle inactivity (hindlimb unloading, immobilization, casting), neural damage (denervation), disease (diabetes, cachexia, obesity), or aging [3,24,37]. It is known to be mainly associated with aging, but it is strictly correlated with a decrease in physical activity [38,39]. As in vivo models for sarcopenia, aged mice, the senescence-accelerated mouse P8, hindlimb unloading, and immobilization model exist [40]. In the present study, we used the hindlimb immobilization model to induce muscle atrophy caused by continuous muscle inactivity.

In the first study, we hydrolyzed whey protein concentrate or acid whey into four different whey protein hydrolysates (AW-H, AW-S, WP-H, and WP-S) and compared their effects on muscle atrophy. We measured the grip strength, muscle weight, CSA, and expressions of markers of muscle protein synthesis and degradation. WP-S increased more the muscle function and mass, which were decreased by immobilization more than the other whey protein hydrolysates (AW-H, AW-S, and WP-H) (Figure 1). This might be because the total BCAA contents of WP-S were the highest compared to other whey protein hydrolysates according to the amino acid composition results (Table 1). Interestingly, WP-S has lower leucine content than AW-H and AW-S and has higher total BCAA contents than AW-H and AW-S. Considering that leucine is widely known to be the most efficient in muscle protein synthesis among BCAAs [41,42], our results showed that the total BCAA contents are more important than leucine content in enhancing skeletal muscle mass. However, except for BCAA, there may be another ingredient of WP-S that has an attenuative function on muscle atrophy. Until now, little is known about whether the ingredients of whey protein hydrolysate stimulate muscle protein synthesis and inhibit muscle protein degradation. Therefore, we are currently trying to find the active component of WP-S by performing in vitro experiments.

Recent studies showed that immobilization significantly reduced muscle strength, mass, and the CSA of muscle fiber [33,43]. Our findings showed a significant decline in those mentioned above and also total protein content. Thus, we confirmed the onset of muscle wasting due to immobilization (Figure 1 and Figure 3). WP-S administration recovered these decreases caused by immobilization, so we verified the efficacy of WP-S against muscle atrophy. Reductions in muscle mass, protein content, and myofiber CSA are caused by an imbalance between muscle protein synthesis and degradation [44]. Some studies suggested that decreased muscle protein synthesis drives muscle wasting [45], but there is still much to be studied. Therefore, we investigated how WP-S administration affected protein synthesis and degradation.

Immobilization suppressed the PI3K/Akt pathway in other studies [46,47] and it was similar in our findings. In Figure 4, the phosphorylation of PI3K and Akt was down-regulated in the IM group and up-regulated in the WP-S treatment groups. Activated Akt increases the net protein content by activating mTOR and inhibiting FoxO3a [15]. The phosphorylation of mTOR, 4E-BP1, and S6K1 was significantly increased by WP-S administration, resulting in the stimulation of muscle protein synthesis. FoxO3a was inactivated through phosphorylation by activated Akt; thus, the expressions of Atrogin-1, MurF1, and Bnip3 were all significantly decreased, resulting in inhibition of muscle protein degradation. The overall rates between muscle protein synthesis and degradation were similar when compared to the downstream protein expressions (4E-BP1 and S6K1 for muscle protein synthesis and Atrogin-1 and MurF1 for muscle protein degradation). In conclusion, WP-S administration increased the net protein content, promoting muscle protein synthesis and inhibiting muscle protein degradation to a similar degree.

Skeletal muscle fibers are classified as slow-twitch (type 1) and fast-twitch (type 2) based on different MyHC expression levels [36,48]. Skeletal muscle expresses four MyHC isoforms: MyHC1 (slow isoform) and MyHC2A, MyHC2X, and MyHC2B (fast isoforms). Type 1 and 2A fibers have higher mitochondria contents and use oxidative metabolism. On the contrary, type 2X and 2B fibers have lower mitochondria contents and use glycolytic metabolism. Muscle inactivity causes type 1 fiber atrophy and shift muscle fiber type from slow (type 1) to fast (type 2A and 2X), whereas aging causes type 2 fiber loss [49,50]. In our study, the total MyHC content was decreased in the IM group and was increased in the WP-S treatment groups in both the soleus and quadriceps (Figure 5). A recent study discovered that MuRF1 binds to the MyHC and degrades it by the proteasome [3]. In Figure 2 and Figure 4, the expression level of MurF1 significantly increased by IM and decreased by WP-S treatment; thus, the expression of total MyHC content showed contrary results against MurF1 expression.

In the MyHC isoform expression, the muscle fiber type shifted from slow to fast by immobilization and it was reversed by WP-S treatment in both the soleus and quadriceps (Figure 5). Given that total MyHC content was decreased in the IM group, it showed that immobilization reduced both slow and fast muscle fibers but strongly affected slow fibers. In addition, WP-S administration increased both slow and fast muscle fibers but enhanced slow fibers heavily. These tendencies showed similarity with muscle weight changes in both the quadriceps and soleus (Figure 1 and Figure 3). Additionally, the increases in fast fibers explained the enhancement in grip strength (Figure 1 and Figure 3) because fast fibers contract strongly using glycolytic metabolism. Considering that WP-S significantly increased type I MyHC in soleus, if we measured aerobic exercise performance ability such as with the treadmill test, WP-S probably increased it significantly. Consequently, WP-S administration restored muscle loss induced by immobilization through enhancing total MyHC expression and regulating its isoform expressions (slow > fast).

## 5. Conclusions

Soluble whey protein hydrolysate (WP-S) significantly increased grip strength, muscle mass, and CSA of muscle fiber reduced by immobilization. WP-S enhanced the net protein content, activating muscle protein synthesis (PI3K/Akt/mTOR pathway) and inhibiting muscle protein degradation (PI3K/Akt/Foxo3a pathway). In addition, WP-S increased total MyHC expression and changed its isoform composition.

## Figures and Tables

**Table 1:** Amino acid composition.

Amino Acids	Contents (mg/g)			
	AW-H	AW-S	WP-H	WP-S
Threonine	35.6	36.5	54.0	54.8
Tyrosine	24.9	25.4	22.3	23.0
Arginine	18.4	18.7	18.9	19.4
Alanine	39.1	38.6	39.3	39.6
Proline	36.8	35.3	39.2	41.7
Lysine	73.2	74.4	69.9	70.9
Histidine	16.5	17.3	16.2	16.1
Isoleucine	36.4	36.5	44.2	45.6
leucine	88.9	88.8	81.3	81.9
Methionine	17.5	17.7	17.4	16.8
Phenylalanine	27.3	28.4	25.8	27.4
Valine	31.1	31.3	40.2	41.9
Glutamine	107.5	111.9	132.8	135.0
Aspartic acid	79.4	81.9	58.9	86.4
Serine	23.5	25.7	42.2	40.9
Glycine	13.4	13.5	12.5	15.8
BCAAs	156.4	156.5	165.7	169.4
Total AA	669.4	681.8	715.0	757.1

**Table 2:** Nutrient contents of soluble whey protein hydrolysate (WP-S).

**Component**		
Energy	kcal/100 g	375.44
Carbohydrate	g/100 g	14.64
Crude Fat	g/100 g	3.72
Crude Protein	g/100 g	70.85
Moisture	%	4.02
Ash	%	6.77

**Table 3:** The primer sequences.

Gene	Forward (5′-3′)	Reverse (5′-3′)
*atrogin-1*	AGA AAG AAA GAC ATT CAG AAC A	GCT CCT TCG TAC TTC CTT
*murf1*	AAG ACT GAG CTG AGT AAC TG	TAG AGG GTG TCA AAC TTC TG
*bnip3*	TTC CAC TAG CAC CTT CTG ATG A	GAA CAC CGC ATT TAC AGA ACA A
*myhc I *	CTC AAG CTG CTC AGC AAT CTA TTT	GGA GCG CAA GTT TGT CAT AAG T
*myhc IIa*	AGG CGG CTG AGG AGC ACG TA	GCG GCA CAA GCA GCG TTG G
*myhc IIx*	GAG GGA CAG TTC ATC GAT AGC AA	GGG CCA ACT TGT CAT CTC TCA T
*myhc IIb*	CAA TCA GGA ACC TTC GGA ACA C	GTC CTG GCC TCT GAG AGC AT
*gadph*	TCG GTG TGA ACG GAT TTG	GGT CTC GCT CCT GGA AGA

## Supplementary Materials

The following are available online at https://www.mdpi.com/2072-6643/12/11/3362/s1, Figure S1: Preparation of whey protein hydrolysates. Figure S2: Experimental design. Figure S3: Total food intake and total protein intake. Figure S4: Body weight. Figure S5: Representative images of H&E stained gastrocnemius section. Figure S6: Liver and kidney function biomarkers. Table S1: Composition of diet (Teklad Global 18% Protein Rodent Diet).
