# Supplementary material for: Soluble Whey Protein Hydrolysate Ameliorates Muscle Atrophy Induced by Immobilization via Regulating the PI3K/Akt Pathway in C57BL/6 Mice
Source: Nutrients. 2020 Nov 1;12(11):3362. doi: 10.3390/nu12113362 (PMC7692342; doi:10.3390/nu12113362)
Supplement: Supplementary file 1 [file nutrients-12-03362-s001.pdf]

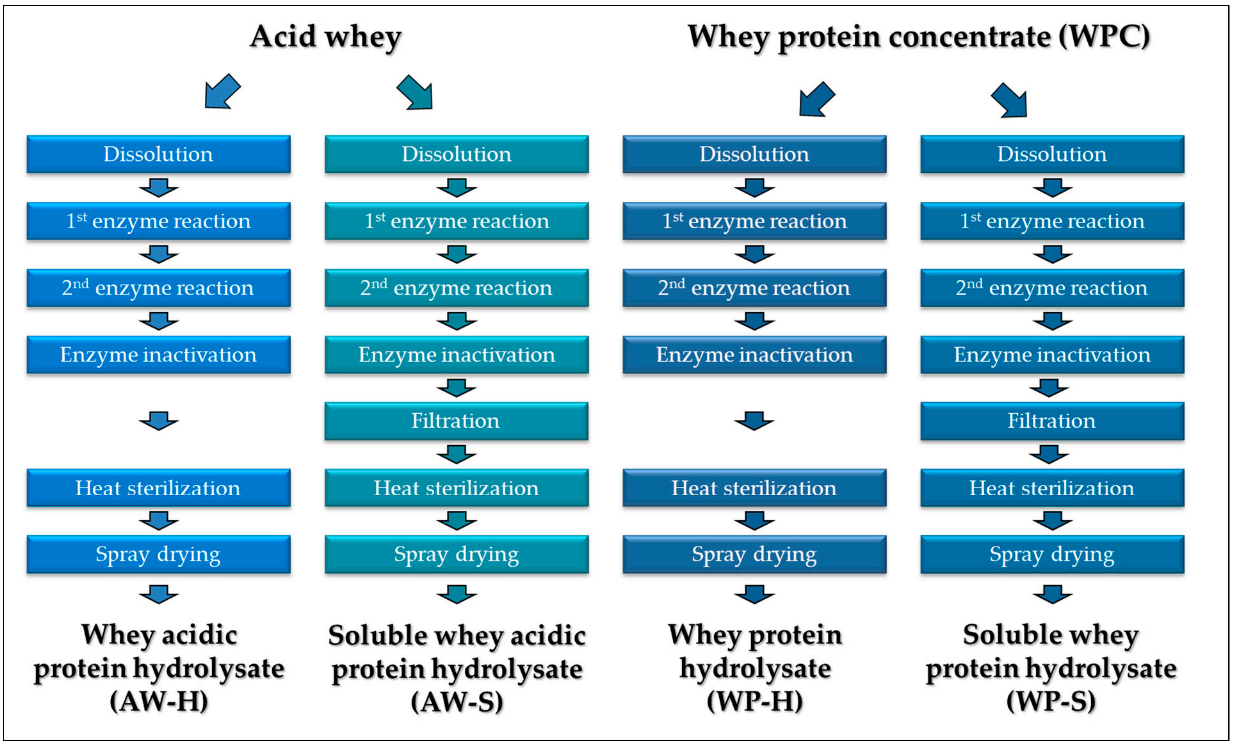

Supplementary Figure S1. Preparation of whey protein hydrolysates

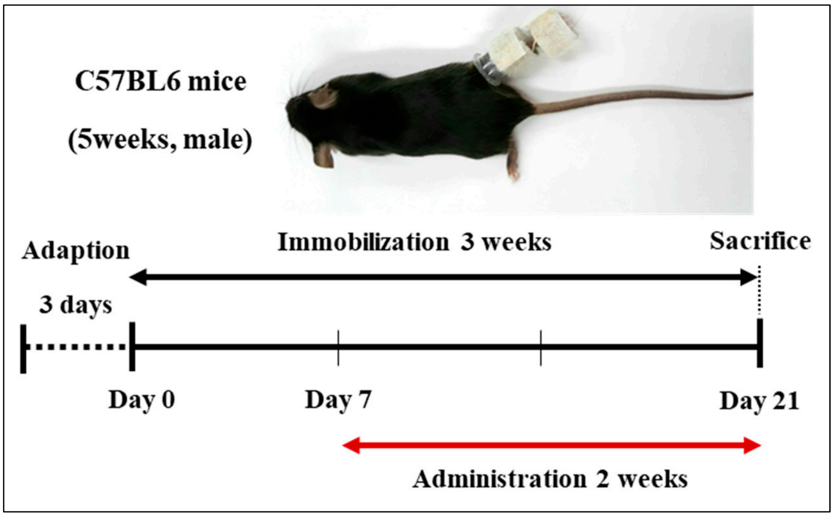

Supplementary Figure S2. Experimental design

Supplementary Table S1. Composition of diet (Teklad Global 18% Protein Rodent Diet)

Macronutrients		
Crude protein	%	18.6
Fat (ether extract)[a]	%	6.2
Carbohydrate (available)[b]	%	44.2
Crude fiber	%	3.5
Neutral Detergent Fiber[c]	%	14.7
Ash	%	5.3
Energy Density[d]	Kcal/g	3.1
Calories from Protein	%	24
Calories from Fat	%	18

Calories from Carbohydrate	%	58

[a]Ether extract is used to measure fat in pelleted diets, while an acid hydrolysis method is required to recover fat in extruded diets. Compared to ether extract, the fat value for acid hydrolysis will be approximately 1% point higher.
[b]Carbohydrate (available) is calculated by subtracting neutral detergent fiber from total carbohydrates.
[c] Neutral detergent fiber is an estimate of insoluble fiber, including cellulose, hemicellulose, and lignin. Crude fiber methodology underestimates total fiber.
[d] Energy density is a calculated estimate of metabolizable energy based on the Atwater factors assigning 4 kcal/g to protein, 9 kcal/g to fat, and 4 kcal/g to available carbohydrate.

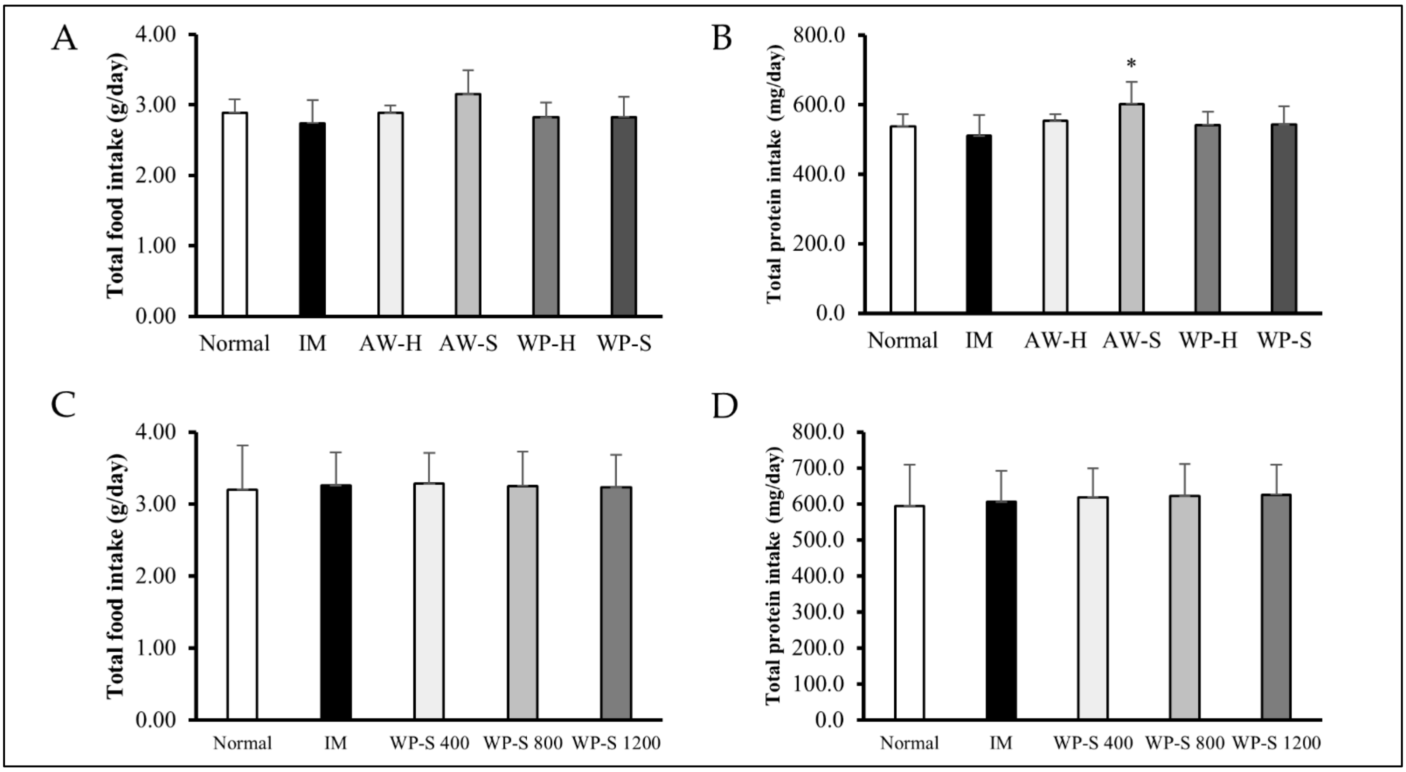

Supplementary Figure S3. Total food intake and total protein intake

(A) Total food intake of the first study. (B) Total protein intake of the first study. (C) Total food intake of the second study. (D) Total protein intake of the second study. Data are expressed as mean ± SD. *p<0.05 versus IM.

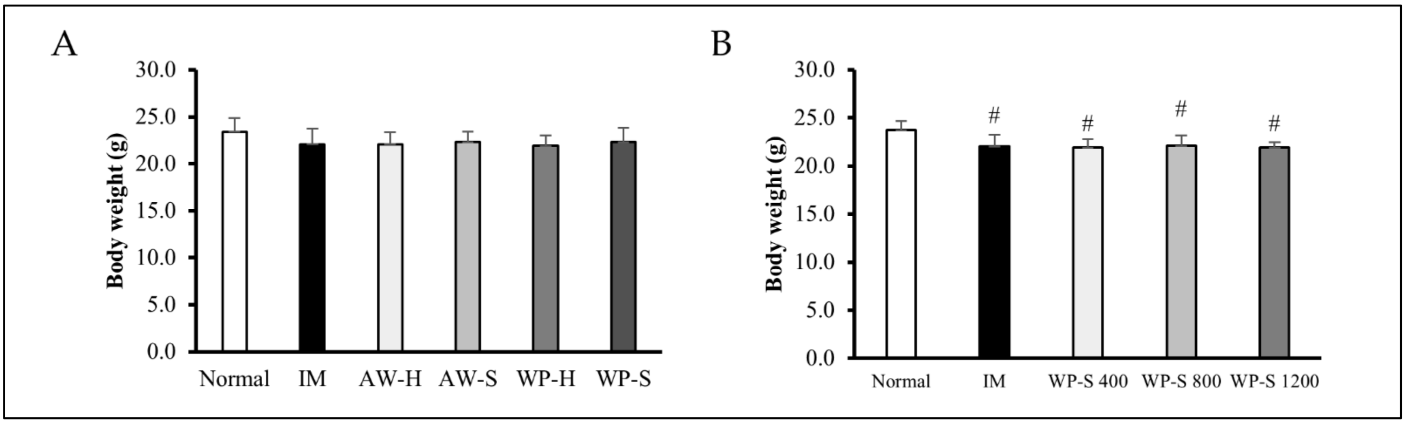

Supplementary Figure S4. Body weight

(A) Body weight of the first study. (B) Body weight of the second study. Data are expressed as mean ± SD. #p<0.05 versus Normal.

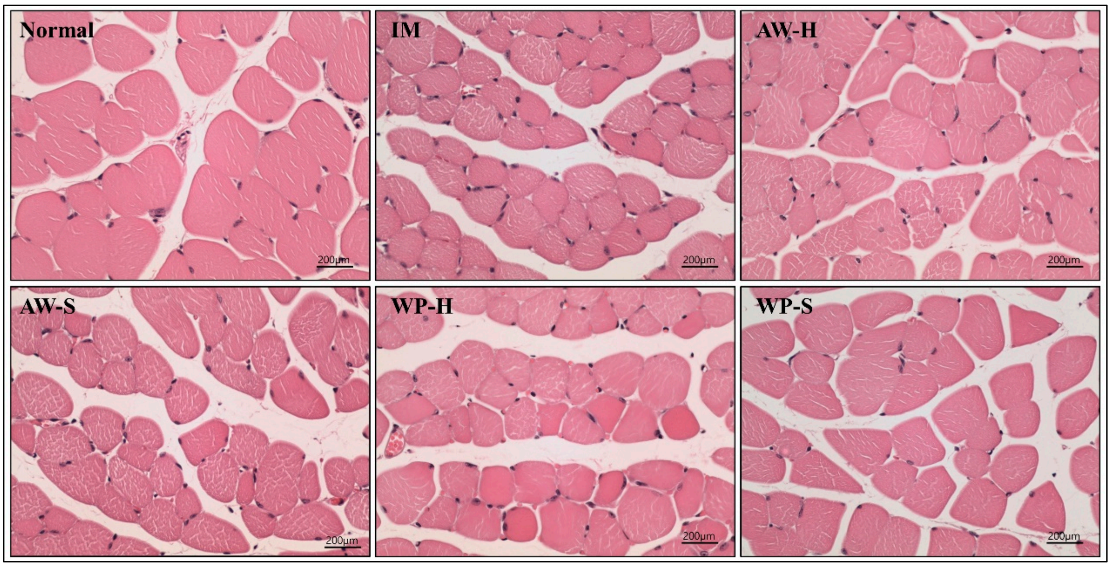

Supplementary Figure S5. Representative images of H&E stained gastrocnemius section

The representative images of H&E stained gastrocnemius sections of the first study (400x, scale bar = 200 μm).

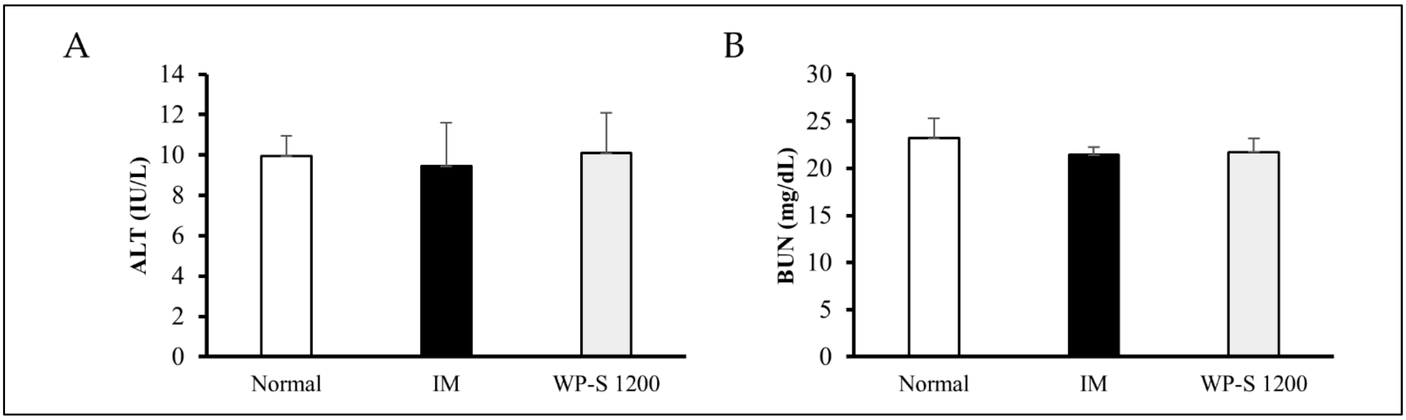

Supplementary Figure S6. Liver and kidney function biomarkers

(A) ALT levels in the serum. ALT is widely known as an indicator of hepatotoxicity in the blood. ALT levels did not show significant differences between groups and were under 20 IU/L, which is regarded as normal in all groups. (B) BUN levels in the serum. BUN is known as a renal function biomarker. BUN levels did not show significant differences between groups and were under 30mg/dL, which is considered normal in all groups. Data are expressed as mean ± SD.
